# Successful Use of Peripheral Nerve Blocks for Emergent Trauma Surgery in a Patient With Perioperative Myocardial Infarction: A Case Report

**DOI:** 10.7759/cureus.116380

**Published:** 2026-09-16

**Authors:** Logan Goddard, Tatiana Salloum, Nicholas Baltera, Daniel Perez

**Affiliations:** 1 Department of Anesthesiology, Perioperative Medicine, and Pain Management, University of Miami, Miami, USA

**Keywords:** anesthesia, case report, leg amputation, myocardial infarction, nerve block, regional anesthesia, st-elevation myocardial infarction (stemi)

## Abstract

Patients with concurrent trauma-related surgical emergencies and ST-segment elevation myocardial infarction (MI) present a unique anesthetic challenge, as damage control surgery and rapid resuscitation must take priority over timely coronary revascularization. During these surgeries, the physiologic insult from general anesthesia can be detrimental to a failing heart, so regional anesthesia techniques and their implications in trauma should be considered. We present a 62-year-old male motorcyclist with hypertension, diabetes, and coronary artery disease and a history of percutaneous coronary intervention who was hit by a car and presented with traumatic partial amputation of the left leg. The patient was brought to the operating room for a left below-knee amputation, and ST-segment elevation in lead II was noted. A 12-lead electrocardiogram showed elevations in II, III, and aVF. We discussed the need for surgery and proceeded with regional anesthesia via left femoral and lateral popliteal sciatic nerve blocks. After surgery, angiography in the cardiac catheterization suite revealed total thrombotic occlusion of the right coronary artery. Cardiology performed thrombectomy, angioplasty, and stent placement. Throughout the hospital course, the patient required multiple incision and drainage procedures, an above-knee amputation, and a second revascularization, all under regional anesthesia. He was eventually discharged to acute rehabilitation. This case underscores the value of damage control surgery and rapid resuscitation, which are paramount for trauma patients with perioperative MIs prior to coronary revascularization. The ability to proceed with regional techniques to avoid the physiologic insult of general anesthesia is invaluable. There are unique considerations for regional anesthesia in trauma patients, such as consent and communication, positioning limitations, coagulopathies, unknown history, and risk of compartment syndrome and infection.

## Introduction

In patients presenting with ST-segment elevation myocardial infarction (STEMI), the American College of Cardiology/American Heart Association (ACC/AHA) guidelines recommend a door-to-balloon time of <90 minutes for patients undergoing primary percutaneous coronary intervention (PCI) [1]. When patients present with emergent surgical needs due to trauma, this timeline can be prolonged, as damage control surgery and rapid resuscitation are paramount. Knowing the type of myocardial infarction (MI) and the effects of different anesthesia techniques is important when determining the anesthetic approach. Inferior wall MIs (IWMIs) are common and generally have a better prognosis than anterior infarctions, although complications such as right ventricular involvement and atrioventricular block can increase mortality [2]. The physiologic insult from general anesthesia could be detrimental to a failing right heart, so regional techniques and their implications in trauma need to be considered.

## Case presentation

This patient is a 62-year-old male motorcyclist with hypertension, diabetes, and coronary artery disease with a history of PCI who was hit by a car. He sustained a traumatic partial amputation of the left lower leg and was brought to the Ryder Trauma Center by Emergency Medical Services. On arrival in the Trauma Resuscitation Unit, his Glasgow Coma Scale score was 14, his airway was patent, his respirations were regular, and his oxygen saturation was in the high 90s on room air. He was borderline hypotensive with mean arterial pressure in the high 60s to low 70s mmHg and tachycardic to the low 100s bpm. He was given 2 units of whole blood with moderate improvement in blood pressure. A whole-body CT scan showed no internal injuries, so he was brought to the operating room (OR) for left below-knee amputation (BKA), and standard anesthesia monitors were attached. The anesthesia team noted ST-segment elevation in lead II and informed the trauma surgeons of the STEMI. A formal electrocardiogram (EKG) showed elevations in leads II, III, and aVF, and interventional cardiology was immediately consulted. The troponin I level, obtained after the STEMI was identified, was markedly elevated at 103 ng/mL.

The team decided to proceed with damage control surgery under monitored anesthesia care (MAC) with regional anesthesia. A formal EKG was performed (Figure 1). The patient was then given 50 mcg of fentanyl and 2 mg of midazolam, and an arterial line was placed. Left femoral and lateral popliteal sciatic nerve blocks were performed under in-plane ultrasound guidance using a 22-gauge, 4-inch short-bevel needle. At each block site, 10 mL of 1% lidocaine and 10 mL of 0.5% ropivacaine (20 mL total per block) were injected (Figures 2-3) for surgical anesthesia, and surgery commenced. Thirty minutes later, the patient became increasingly hypotensive, and a norepinephrine infusion was started, 325 mg of aspirin was given orally, and one unit of packed red blood cells was transfused. A bedside transthoracic echocardiogram with cardiology showed hypokinesis of the inferior and posterior left ventricular (LV) walls and the right ventricle.

**Figure 1:**
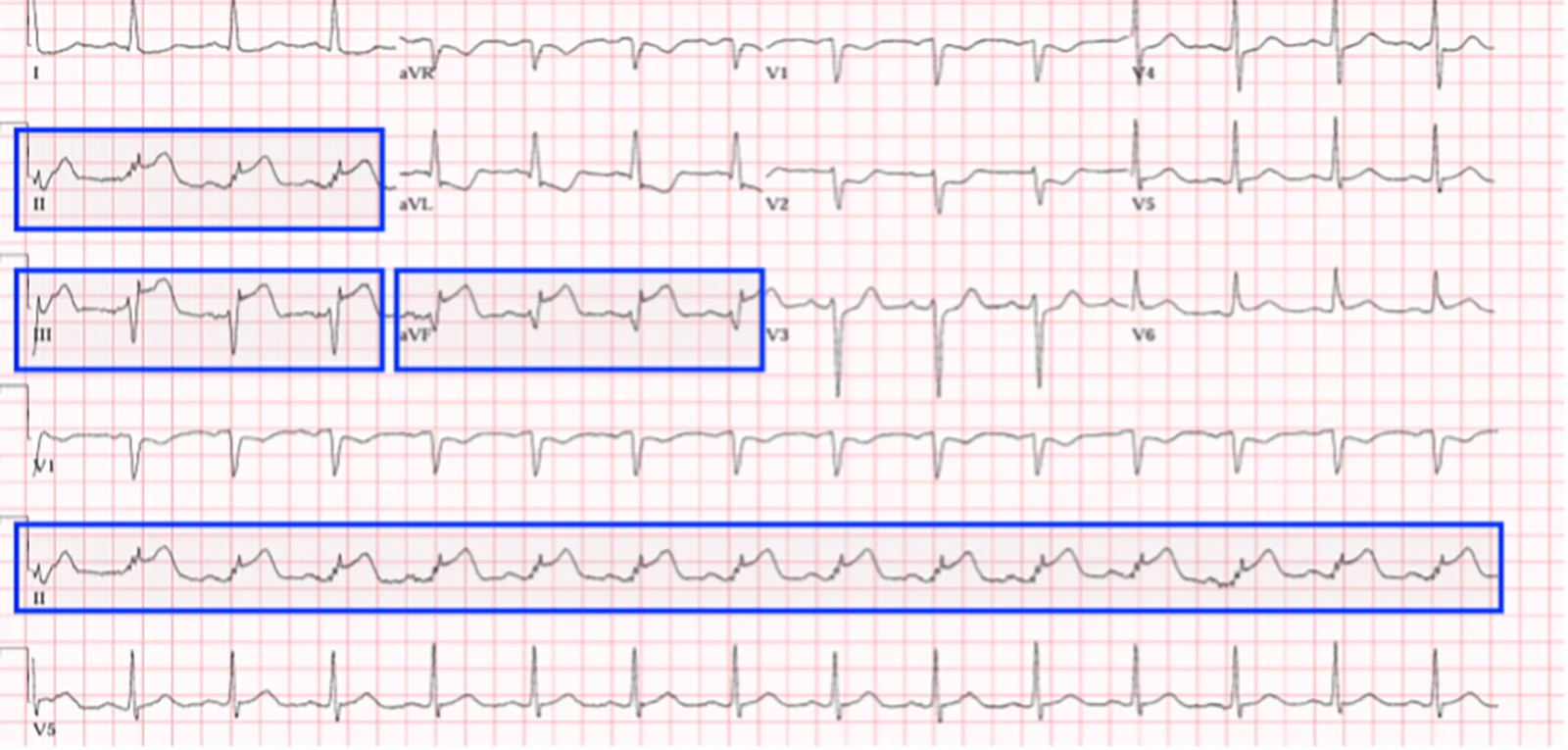
Intraoperative 12-lead EKG showing ST-segment elevations (blue boxes) EKG: electrocardiogram

**Figure 2:**
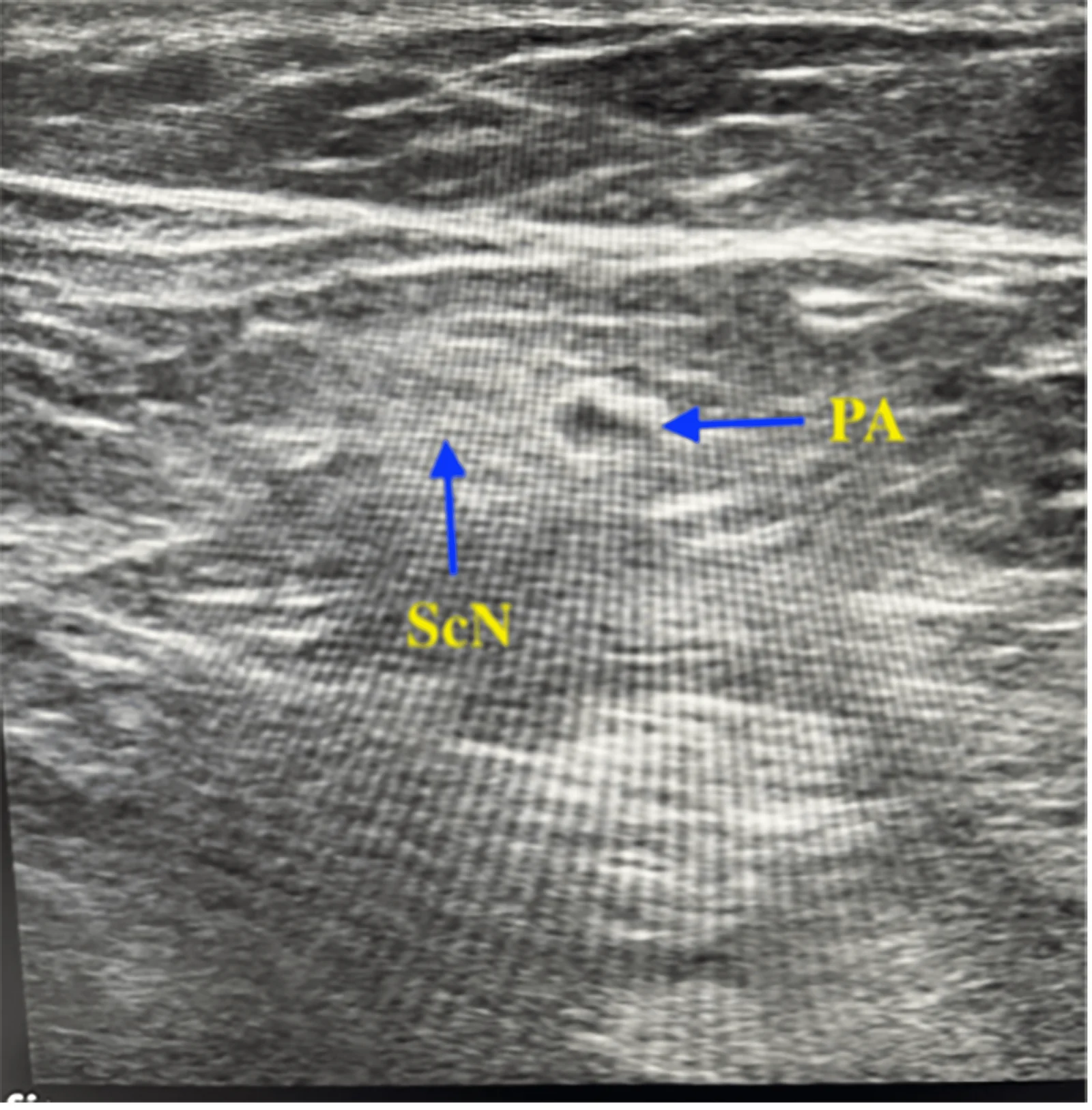
Ultrasound of lateral popliteal sciatic nerve block ScN: sciatic nerve, PA: popliteal artery (blue arrows)

**Figure 3:**
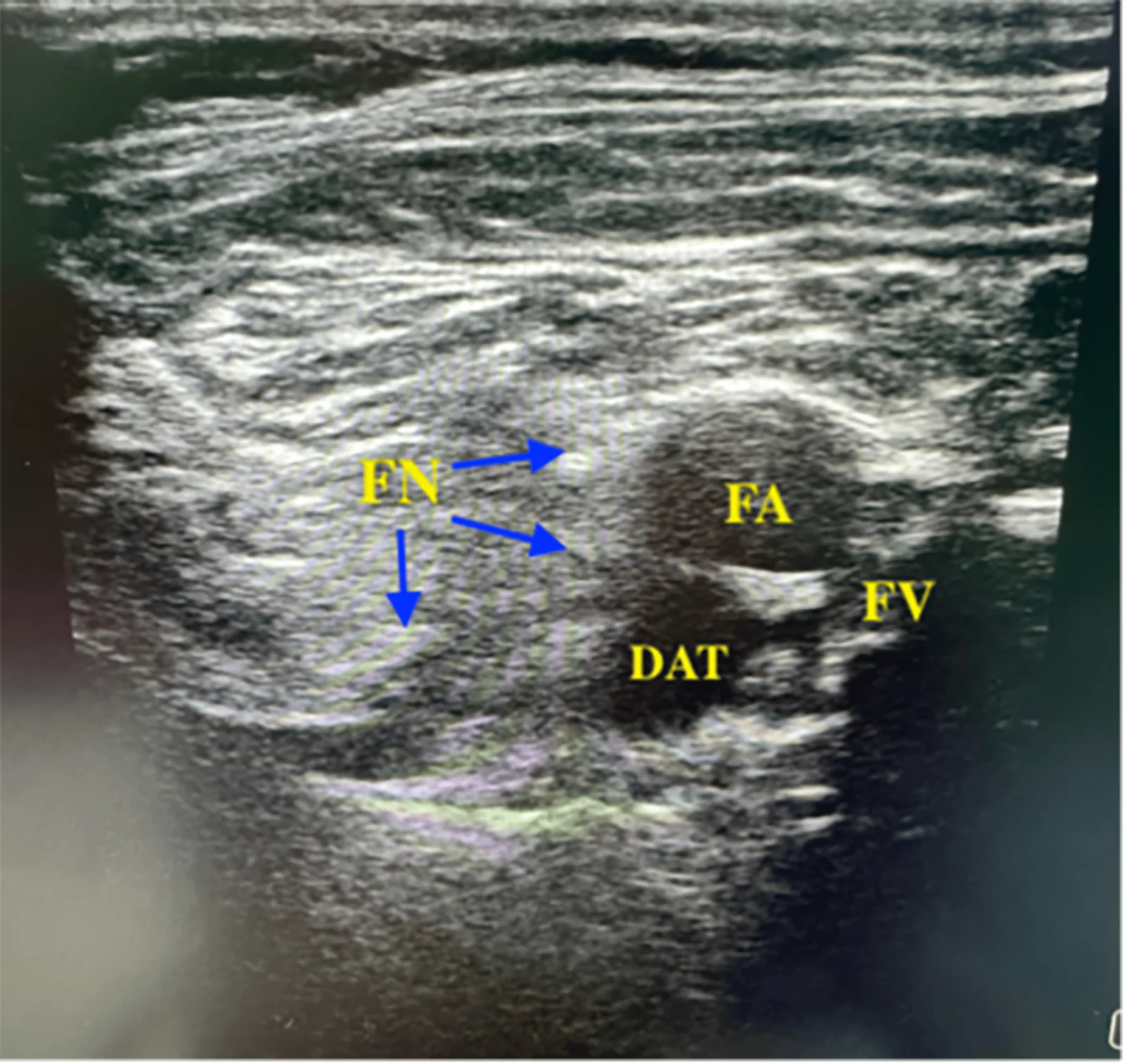
Ultrasound of femoral nerve block FN: femoral nerve (blue arrows), FA: femoral artery, FV: femoral vein, DAT: deep artery of the thigh

The surgery was completed in under 90 minutes, and the patient was taken to the cardiac catheterization laboratory. The coronary angiography revealed total thrombotic occlusion of the right coronary artery (RCA) (the dominant artery in this patient). Interventional cardiology placed an intra-aortic balloon pump (IABP) and performed aspiration thrombectomy, angioplasty, and stent placement, restoring perfusion. The patient was started on clopidogrel and aspirin for the remainder of his hospital course.

The patient was brought to the intensive care unit (ICU), and the IABP was removed the following day. Troponin levels trended downward postoperatively. While in the ICU, the acute pain service placed a left lateral popliteal sciatic catheter for postoperative analgesia. However, three days later, the patient developed signs of infection at the left BKA margins. He was brought to the OR for incision and drainage (I&D) of the left lower extremity with an anesthetic plan for MAC, femoral nerve block, and a bolus of the lateral popliteal catheter. The surgery was completed without complication, and he was brought to the ICU, where the popliteal catheter was removed due to infection concerns.

Within five days of ICU admission, the patient developed fever and tachycardia with further signs of BKA stump infection; he was taken to the OR for above-knee amputation with orthopedics. MAC was administered, and femoral, lateral femoral cutaneous, classic sciatic, and obturator nerve blocks were performed at the groin and proximal thigh/gluteal region, anatomically remote from the infected BKA stump. Shortly after this procedure, anesthesia once again noticed lead II ST elevations, and the patient was brought to the cath lab, where a complete rethrombosis of his RCA was discovered. The team performed a second aspiration, embolectomy, and stent placement, restoring perfusion. The patient underwent one further I&D under regional anesthesia and was discharged to acute rehab after 1.5 months in the hospital.

## Discussion

Damage control surgery and rapid resuscitation are paramount for trauma patients with perioperative MIs and emergent surgical needs prior to coronary revascularization [3]. However, trauma patients can have difficult airways, increased risk for aspiration, and multiple comorbid conditions that put them at higher risk for general anesthesia. Patients with IWMIs require careful treatment with IV fluids and vasopressors to maintain preload, and nitrates and beta blockers often must be avoided, further increasing the risks of general anesthesia [4]. General anesthesia decreases systemic vascular resistance, reducing preload. In this case, prophylactic antibiotics were not administered prior to the initial emergent amputation, as resuscitation and hemorrhage control took priority; this may have contributed to the subsequent surgical site infection at the amputation stump.

General anesthesia typically necessitates the use of positive pressure ventilation, which causes a multitude of cardiovascular effects, such as a decrease in preload, increased pulmonary vascular resistance and thus an increase in right ventricular (RV) afterload, and a small decrease in LV afterload with improvement in LV ejection fraction (EF), which studies suggest is most pronounced in patients with heart failure with reduced EF. These physiologic effects of general anesthesia could be detrimental for a patient with an IWMI with right-sided involvement. Regional anesthesia provides an excellent alternative in these cases, with its analgesic benefits improving respiratory mechanics. This can decrease the risk of hypoxia, hypercarbia, and pulmonary vasoconstriction, which is especially important in patients with IWMIs and poor right heart function.

Several aspects of regional anesthesia make it ideal for emergent surgeries in patients with MI. Multiple studies have shown that successful peripheral nerve blocks can provide excellent analgesia without disrupting hemodynamics or respiratory status [5,6]. By avoiding large swings in blood pressure and heart rate, the risk of hypoperfusion is mitigated, potentially reducing morbidity and mortality. Furthermore, the long-term effects of early nerve blockade can provide better postoperative analgesia and reduce the incidence and severity of chronic post-traumatic pain syndromes [7]. Peripheral nerve blocks also offer advantages over neuraxial anesthesia; one recent study showed a lower incidence of hypotension with peripheral nerve blocks [8]. Furthermore, neuraxial anesthesia carries a greater risk of hematoma in anticoagulated patients than most peripheral nerve blocks [9]. With these benefits in mind, a trauma center's ability to use regional techniques to avoid the physiologic insult of general anesthesia is invaluable.

Regional anesthesia does not come without risks, and there are unique considerations for these anesthetic techniques, particularly in trauma patients. These patients often require rapid resuscitation to preserve life, restricting the time available to perform regional anesthesia [10-12]. Positioning issues can also arise as patients may need to remain supine to maintain hemodynamic stability, preventing posterior access for posterior techniques [10]. Cervical collars and patient pain may also impede proper positioning [10,13]. Trauma patients often have impaired consciousness, which can compromise their ability to communicate effectively and create challenges in obtaining informed consent [14]. Limited communication also raises concerns about increased risk of iatrogenic injury since patients cannot report any symptoms that arise during regional anesthetic administration [15]. In addition to unknown past medical history and baseline nerve function, trauma patients may also have sustained nerve injuries that increase the likelihood of permanent injury during treatment [16,17]. Trauma patients also have a higher risk of coagulopathy due to trauma-related factors, and their preinjury anticoagulation status may be unknown [16]. Bleeding consequences from neuraxial anesthesia could lead to paralysis or life-threatening hemorrhage due to undetected bleeding during deep blocks. Infection risk is similarly elevated in this patient population, as impaired immunity and damaged physical barriers enable pathogens to flourish [18].

No clear consensus exists on whether regional or general anesthesia leads to better outcomes. For example, the REGAIN trial, published in the New England Journal of Medicine in 2021, was a randomized superiority trial with 1,600 patients that compared spinal anesthesia with general anesthesia in previously ambulatory patients undergoing surgery for hip fractures [19]. They assessed outcomes of mortality, ability to walk, time to discharge, and delirium within 60 days of surgery. They found no significant difference between the two anesthetic techniques. A 2020 retrospective study in the International Journal of Surgery included 90,785 non-cardiac and non-neurosurgery patients [20]. This propensity score-matched cohort study found lower rates of 30-day mortality and postoperative ICU admission in patients who underwent regional anesthesia versus general anesthesia. These two studies show differing outcomes, and more research is needed.

## Conclusions

Despite the lack of direct evidence demonstrating the type of anesthesia that leads to the best outcomes for trauma patients, there is consensus that regional techniques should be integrated into multimodal pain management. When performed by experienced providers under ultrasound guidance, regional techniques provide safe, effective pain management for trauma patients. Given the potential infection risk associated with catheter-based regional techniques in trauma patients, infection-prevention measures should be carefully considered in this population. This case further supports the value of regional anesthesia. Each patient should have an individualized anesthetic plan that considers medical comorbidities, current physiologic status, surgical urgency, and the specific physiologic effects, risks, and benefits of regional or general anesthesia before proceeding with surgery. In this case of perioperative MI, regional anesthesia provided safe, effective analgesia for multiple major surgeries.

## References

[1] Rao SV, O'Donoghue ML, Ruel M (2025). 2025 ACC/AHA/ACEP/Naemsp/SCAI guideline for the management of patients with acute coronary syndromes: a report of the American College of Cardiology/American Heart Association Joint Committee on clinical practice guidelines. J Am Coll Cardiol.

[2] Aguiar Rosa S, Timóteo AT, Ferreira L (2018). Complete atrioventricular block in acute coronary syndrome: prevalence, characterisation and implication on outcome. Eur Heart J Acute Cardiovasc Care.

[3] Thompson A, Fleischmann KE, Smilowitz NR (2024). 2024 AHA/ACC/ACS/ASNC/HRS/SCA/SCCT/SCMR/SVM guideline for perioperative cardiovascular management for noncardiac surgery: a report of the American College of Cardiology/American Heart Association Joint Committee on clinical practice guidelines. Circulation.

[4] Damluji AA, van Diepen S, Katz JN (2021). Mechanical complications of acute myocardial infarction: a scientific statement from the American Heart Association. Circulation.

[5] Kim NY, Lee KY, Bai SJ, Hong JH, Lee J, Park JM, Kim SH (2016). Comparison of the effects of remifentanil-based general anesthesia and popliteal nerve block on postoperative pain and hemodynamic stability in diabetic patients undergoing distal foot amputation: a retrospective observational study. Medicine (Baltimore).

[6] Huaguo Y, Kang S, Hu L, Zhou H (2024). Advancing pain management for extremity trauma: the evolution of ultrasound-guided nerve blocks for patients in the supine position in trauma centers. Eur J Trauma Emerg Surg.

[7] Bansal L, Attri JP, Verma P (2016). Lower limb surgeries under combined femoral and sciatic nerve block. Anesth Essays Res.

[8] Lai HY, Foo LL, Lim SM (2020). The hemodynamic and pain impact of peripheral nerve block versus spinal anesthesia in diabetic patients undergoing diabetic foot surgery. Clin Auton Res.

[9] Kietaibl S, Ferrandis R, Godier A (2022). Regional anaesthesia in patients on antithrombotic drugs: Joint ESAIC/ESRA guidelines. Eur J Anaesthesiol.

[10] Saranteas T, Koliantzaki I, Savvidou O, Tsoumpa M, Eustathiou G, Kontogeorgakos V, Souvatzoglou R (2019). Acute pain management in trauma: anatomy, ultrasound-guided peripheral nerve blocks and special considerations. Minerva Anestesiol.

[11] Torrie AM (2022). Regional anesthesia and analgesia for trauma: an updated review. Curr Opin Anaesthesiol.

[12] Tobin JM, Barras WP, Bree S (2018). Anesthesia for trauma patients. Mil Med.

[13] Patel MB, Humble SS, Cullinane DC (2015). Cervical spine collar clearance in the obtunded adult blunt trauma patient: a systematic review and practice management guideline from the Eastern Association for the Surgery of Trauma. J Trauma Acute Care Surg.

[14] Lin YK, Liu KT, Chen CW, Lee WC, Lin CJ, Shi L, Tien YC (2019). How to effectively obtain informed consent in trauma patients: a systematic review. BMC Med Ethics.

[15] Bage T, Power DM (2021). Iatrogenic peripheral nerve injury: a guide to management for the orthopaedic limb surgeon. EFORT Open Rev.

[16] Dattatri R, Jain VK, Iyengar KP, Vaishya R, Garg R (2021). Anaesthetic considerations in polytrauma patients. J Clin Orthop Trauma.

[17] Slade IR, Samet RE (2018). Regional anesthesia and analgesia for acute trauma patients. Anesthesiol Clin.

[18] Mas-Celis F, Olea-López J, Parroquin-Maldonado JA (2021). Sepsis in trauma: a deadly complication. Arch Med Res.

[19] Neuman MD, Feng R, Carson JL (2021). Spinal anesthesia or general anesthesia for hip surgery in older adults. N Engl J Med.

[20] Luo X, Liu B, Li F (2020). The relationship between anesthetic technique and thirty-day mortality in patients undergoing noncardiac- and nonneurosurgery: a retrospective, propensity score-matched cohort study. Int J Surg.

